# Phase 1 study of cardiac safety of TAS-102 in patients with advanced solid tumors

**DOI:** 10.1007/s00280-016-3031-9

**Published:** 2016-05-05

**Authors:** Johanna C. Bendell, Manish R. Patel, Kenichiro Yoshida, Jabed Seraj, Racquel Weaver, Charlotte Lemech, Thomas G. Todaro, Shubham Pant, Hendrik-Tobias Arkenau

**Affiliations:** GI Oncology Research, Drug Development Unit, Sarah Cannon Research Institute, 250 25th Avenue N, Suite 100, Nashville, TN 37203 USA; Florida Cancer Specialists, 600 N Cattlemen Rd, Suite 200, Sarasota, FL 34232 USA; Taiho Oncology Inc, 101 Carnegie Center, Suite 101, Princeton, NJ 08540 USA; Sarah Cannon Research Institute UK, 93 Harley Street, London, W1G 6AD UK; Medpace, 5375 Medpace Way, Cincinnati, OH 45227 USA; Peggy and Charles Stephenson Cancer Center, Science Center, 800 NE 10th Street, Suite 2500, Oklahoma City, OK 73104 USA

**Keywords:** 5-Fluorouracil, Cardiac, QT interval, TAS-102, Tipiracil, Trifluridine

## Abstract

**Purpose:**

TAS-102 is a novel oral agent combining the antineoplastic thymidine-based nucleoside analogue, trifluridine, and the thymidine phosphorylase inhibitor, tipiracil (molar ratio 1:0.5). TAS-102 has shown good activity in refractory metastatic colorectal cancer with acceptable safety. No QT prolongation was seen in clinical studies. This study aimed to investigate TAS-102 cardiac safety for regulatory requirements.

**Methods:**

This was a phase 1, non-randomized study in adults with advanced solid tumors. Intensive QT assessments were conducted at baseline, placebo, and following single and multiple doses of TAS-102 during a 28-day cycle.

**Results:**

Following single- and multiple-dose administration (*N* = 30), the upper bounds of the one-sided 95 % confidence intervals for the difference between TAS-102 and placebo in time-matched baseline-subtracted 12-lead Holter QT intervals did not exceed 20 ms at any prespecified time point. One patient had a change from baseline in QTcI interval ≥60 ms, and one patient had a QTcI interval >500 ms following multiple-dose TAS-102 administration. No patient had an uncorrected QT, QTcF, or QTcB interval >500 ms. Based on the exposure-response analysis between TAS-102 plasma concentrations and the placebo-adjusted QTc intervals, none of the upper bounds of the one-sided 95 % prediction intervals exceeded 20 ms. There were no significant morphological changes for T or U waves. No cardiovascular AEs were reported in cycle 1. Across all cycles, no patient experienced an AE of ventricular tachycardia, ventricular fibrillation, syncope, or seizure.

**Conclusions:**

There was no clinically relevant relationship between TAS-102 plasma concentrations and QTc interval; TAS-102 had no clinically relevant effects on cardiac repolarization.

**Clinical trials:**

ClinicalTrials.gov study number: NCT01867879.

## Introduction

Nucleoside inhibition has played an important role in the treatment of cancer. Notably fluoropyrimidines, including 5-fluorouracil and its derivatives, have been an important class of medications used in oncology for decades [1].

TAS-102 is a novel oral agent that combines trifluridine (FTD), a thymidine-based nucleoside analogue, plus tipiracil hydrochloride (TPI), in a molar ratio of 1:0.5 [2, 3]. TPI has been shown to be a potent and specific inhibitor of thymidine phosphorylase in animal models and human tumor cells, and allows sustained FTD concentrations to be maintained after oral administration [4].

TAS-102 has been investigated in preclinical studies, with no identified effect on cardiac ventricular repolarization, using the in vitro hERG assay. Similarly, in conscious monkeys, TAS-102 showed no effects on blood pressure, heart rate, and electrocardiographic parameters at a dose that produced a maximum plasma concentration (C_max_) of FTD and TPI similar to that observed in humans with the anticipated clinical dose of TAS-102.

In humans, TAS-102 has been investigated in phase 1 dose-finding and food-effect studies and clinical studies in patients with refractory metastatic colorectal cancer [3, 5–7]. There has been no evidence of an effect of TAS-102 on cardiac events in a phase 1 (*N* = 27) or phase 3 clinical study (*N* = 800) [6, 7].

The present study was conducted to investigate the effect of TAS-102 on cardiac repolarization after single-dose and multiple-dose administration; to evaluate the cardiac safety profile of TAS-102; and to evaluate the relation between TAS-102 pharmacokinetics and its effect on cardiac repolarization.

## Materials and methods

### Study design

The study was a phase 1, non-randomized study, which was open-label except for a single, patient-blinded dose of placebo on the morning of day −1 to control for individual variability in electrocardiogram (ECG) responses. Given the cytotoxic nature of TAS-102, the design of the study was chosen to comply as closely as ethically possible to the International Conference on Harmonisation E14 Guideline (The Clinical Evaluation of QT/QTc Interval Prolongation and Proarrhythmic Potential for Non-antiarrhythmic Drugs) [8, 9]. Although not a true thorough QT/QTc study, the study included intensive QT assessments during a full-time-matched baseline day and full-day assessments following administration of a single dose of placebo and single and multiple doses of TAS-102.

The cardiac safety evaluation took place during cycle 1 of treatment over 28 days and was the first of two parts of the overall study. The second part was an extension phase consisting of the same 28-day cycle, which was repeated until the patient met any of the treatment discontinuation criteria. Efficacy and safety assessments were performed during the second part of the study and are not included in this report, which only includes the cardiac safety results of cycle 1.

### Endpoints

The primary endpoint was the time-matched difference in QTcI interval (the QT interval corrected for heart rate using a patient-specific correction) between TAS-102 and placebo at each time point. QTcI was determined as *QTcI* = *QT/RR*^*βi*^, where *βi* is the patient-specific correction factor computed from a log-linear model *log(QT)* = *α*_*I*_ + *β*_*i*_*x log(RR)* using information obtained at baseline (day −2) from each individual patient *i*. Secondary endpoints were the relation between TAS-102 pharmacokinetics, and its effect on cardiac repolarization and qualitative ECG changes from baseline based on evaluation of 12-hour Holter recordings with extracted 12-lead ECG recordings.

### Patient population

The study population consisted of male and female patients ≥18 years of age with histologically or cytologically confirmed advanced solid tumors (other than breast cancer) for whom no standard therapy existed. Major inclusion criteria included having received ≤5 prior cytotoxic cancer therapies; Eastern Cooperative Oncology Group performance status 0 or 1 on day −2; ability to take medications orally without a feeding tube; corrected QT interval using Bazett’s correction (QTcB) ≤450 ms on resting ECG; and adequate organ function.

Major exclusion criteria were major surgery or extended field radiation within 4 weeks; baseline heart rate >100 beats per minute (bpm) or <50 bpm; extended field radiation within 4 weeks or limited field radiation within 2 weeks; any anticancer therapy within 3 weeks (mitomycin within 5 weeks); serious acute or chronic illness or medical or psychiatric condition(s) or laboratory abnormality that may have increased the risk associated with study participation or may have interfered with interpretation of study results; and concomitant use of a medication known to affect the QT interval or be arrhythmogenic.

The study was conducted, and informed consent was obtained, according to the ethical principles originating in the Declaration of Helsinki (2008) and in accordance with Title 21 CFR 312.50 through 312.70, the International Council for Harmonisation Harmonised Tripartite Guidelines for Good Clinical Practice, and local and national laws and regulations relevant to the use of investigational therapeutic agents. The protocol was approved by the local institutional review board.

### Procedures

**Fig. 1:**
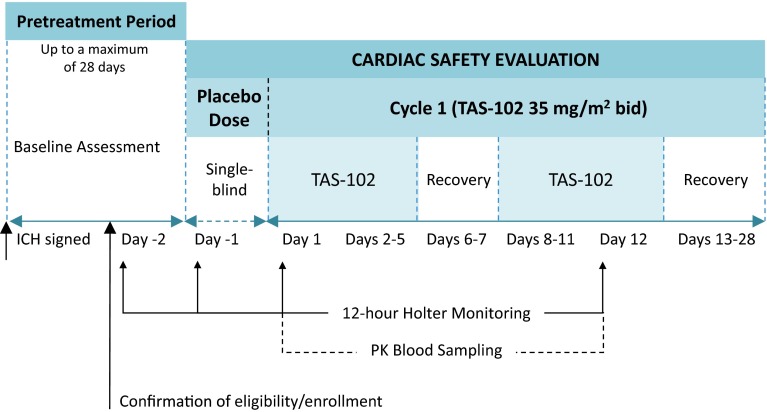
Study design of cardiac safety evaluation. ICH, International Council for Harmonisation; *PK* pharmacokinetics

On day −1, the day prior to the first dose of TAS-102, all patients received a single, patient-blinded dose of placebo in the morning corresponding to a 35 mg/m^2^ dose of TAS-102 based on body surface area (Fig. 1). On days 1 through 5 and 8 through 12, patients received TAS-102 35 mg/m^2^ twice daily within 1 h after completing a meal. On days 1 and 12, the second dose of the day was administered 12 h after the morning dose to allow for collection of a 12-hour pharmacokinetic sample.

Dose reductions for Grade ≥3 non-hematologic drug-related toxicity were allowed in 5 mg/m^2^ steps to a minimum dose of 20 mg/m^2^. The dose of TAS-102 was held for neutrophils <500/mm^3^ or platelets <50 000/mm^3^.

Twelve-hour Holter ECG recordings were obtained within 48 h prior to the first active dose of TAS-102 (day −2), as well as following administration of placebo on day −1 and TAS-102 on day 1 (single-dose) and day 12 (multiple-dose). Twelve-lead ECG recordings were analyzed at 0, 15, and 30 min, and 1, 2, 4, 6, 8, 10, and 12 h postdose. At each time point, the following were measured: respiratory rate, pulse rate, QRS interval, heart rate, and uncorrected and corrected QT interval. Twelve-lead ECGs were obtained at screening and prior to the morning dose on days 1 and 12. Three 10-second 12-lead ECG tracings were extracted within a 5-minute period at each time point. The analysis included each of the three ECGs from a triplicate (three ECGs at each time point) as a single observation. Each of the three ECGs at a single time point was interpreted as normal or abnormal by a single, central, independent cardiologist blinded to treatment, time, and day. The global median beat was prespecified as the lead for interval measurements, and the same lead was used for baseline and postbaseline assessments.

Blood samples were collected approximately 5 min after the corresponding nominal time points for extraction of digital ECG data to measure plasma concentrations of TAS-102 components (FTD and TPI) and metabolite (trifluoromethyl-2,4(1*H*,3*H*)-pyrimidinedione [FTY]).

### Statistics

Triplicate ECG values obtained at each time point were averaged for analysis of the primary endpoint. Descriptive statistics were provided at each time point for QTcI, QTcB, QTcF, QT, PR, QRS, and RR intervals, and heart rate for baseline (day −2) and days −1, 1, and 12, and the corresponding change from baseline. For each postdose scheduled time point of ECG collection, the baseline-subtracted QTcI interval was analyzed using a repeated measure analysis of variance (ANOVA), separately for ECGs assessed during days 1 and 12 of TAS-102 dosing compared with placebo. The model includes the factors of treatment, time, and treatment by time interaction. The measurements within each patient’s treatment were treated as repeated measures. The compound symmetry covariance was used. The upper bounds of the one-sided 95 % confidence interval and the point estimate were obtained for the differences in time-matched baseline-subtracted QTc between TAS-102 and placebo, separately for day 1 and day 12. Similar ANOVA models were applied to analyze QTcF and QTcB intervals. The non-inferiority margin for this study was 20 ms. A sequential test procedure was used to assess whether the placebo-corrected, time-matched differences in QTc exceeded 20 ms at any time point on days 1 and 12. The required parameter and intra-patient error term standard deviation (12.3 ms) were adopted from Zhang et al. to compute the sample size [10]. If the true difference in the QTcI between TAS-102 and placebo was ≤5 ms at all measurement time points, 30 evaluable patients would ensure at least 90 % power to conclude that TAS-102 was non-inferior to placebo with respect to the effect on QTcI interval prolongation at a 5 % significance level.

The number and percentage of patients having maximum changes from baseline in the categories of <20, ≥20 and <30, ≥30 and <60, and ≥60 ms were presented for the following ECG parameters: QTcI, QTcF, and QTcB by time point for each assessment day. A similar tabulation was provided for the maximum individual postdose absolute value in the categories of >450 and ≤480 ms, >480 and ≤500 ms, and >500 ms. The number and percentage of patients with normal or abnormal findings for T wave and U wave were summarized. The relation between plasma concentrations of TAS-102 and the change from baseline in QTc adjusted by placebo was quantified using a linear mixed-effect model approach. All patients who had paired ECG and plasma concentrations for FTD, FTY, and TPI were included in the analysis.

## Results

### Disposition of subjects

A total of 66 patients signed informed consent, of which 22 failed to meet eligibility criteria. The 44 patients who were treated comprise the safety population. Fourteen of the 44 patients were excluded, leaving 30 to comprise the cardiac safety study population. Reasons for exclusion were missing and/or inadequate ECG data (9), taking a prohibited medication (3), low baseline electrolytes that may have affected QT time (2), and missed or reduced drug dosing/non-compliance (1); one patient had two reasons for exclusion. The demographic and baseline characteristics of the safety and cardiac safety populations were similar (Table 1).

**Table 1:** Demographic and baseline characteristics of the cardiac safety population

Parameter	Safety population (*n* = 44)	Cardiac safety population (*n* = 30)
Age, year		
Mean	59.0	58.9
SD	8.86	8.10
Median	59.0	58.0
Minimum, maximum	39, 78	41, 77
Gender, *n* (%)		
Male	22 (50.0)	15 (50.0)
Female	22 (50.0)	15 (50.0)
Race, *n* (%)		
White	38 (86.4)	26 (86.7)
Black, of African heritage	3 (6.8)	2 (6.7)
Asian	2 (4.5)	2 (6.7)
American Indian or Alaskan Native	1 (2.3)	0
Ethnicity, *n* (%)		
Hispanic or Latino	1 (2.3)	1 (3.3)
Not Hispanic or Latino	43 (97.7)	29 (96.7)
Body surface area, m^2^		
Mean	1.92	1.91
SD	0.283	0.253
Median	1.91	1.88
Minimum, maximum	1.44, 2.66	1.48, 2.34
ECOG performance status, *n* (%)		
0	29 (65.9)	21 (70.0)
1	15 (34.1)	9 (30.0)
Cancer type, *n* (%)		
Cervical	2 (4.5)	1 (3.3)
Colon	32 (72.7)	23 (76.7)
Rectal	1 (2.3)	1 (3.3)
Esophageal	2 (4.5)	0
Pancreatic	3 (6.8)	3 (10.0)
Renal	2 (4.5)	1 (3.3)
Uterine	1 (2.3)	0
Other	1 (2.3)	1 (3.3)
Prior surgery/biopsy related to cancer,^a^ *n* (%)		
Yes (excludes patients with biopsy only)	35 (79.5)	23 (76.7)
Primary	27 (61.4)	16 (53.3)
Other	19 (43.2)	13 (43.3)
Biopsy	34 (77.3)	24 (80.0)
No	0	0
Prior radiotherapy		
Yes	18 (40.9)	12 (40.0)
No	26 (59.1)	18 (60.0)
Prior systemic cancer therapy,^b^ *n* (%)		
Yes	44 (100)	30 (100)
Intent of prior systemic cancer therapy,^a^ *n* (%)		
Neoadjuvant	8 (18.2)	5 (16.7)
Adjuvant	15 (34.1)	8 (26.7)
Metastatic	40 (90.9)	28 (93.3)
Number of prior regimens,^b^ *n* (%)		
1	1 (2.3)	0
2	10 (22.7)	7 (23.3)
3	7 (15.9)	5 (16.7)
≥4	26 (59.1)	18 (60.0)

*SD* standard deviation; *ECOG* Eastern Cooperative Oncology Group

^a^Patients with multiple levels are counted in each applicable category

^b^Included all prior systemic therapies (neoadjuvant, adjuvant, metastatic)

The cardiac safety population consisted of all patients who took part of any dose of the study treatment and who had at least 50 % time-matched triplicate ECG measurements on days −2, −1, 1, and 12. Patients could not have taken prohibited concomitant medications, missed or reduced doses of TAS-102, not been <100 % compliant with TAS-102, or had a major protocol deviation. The cardiac safety population was utilized for analysis of all QTc data.

The median dose intensity was 162.30 mg/m^2^/week and the median relative dose intensity, i.e., the ratio of actual dose intensity to planned dose intensity, was 0.93.

### QT interval

#### QTcI

**Fig. 2:**
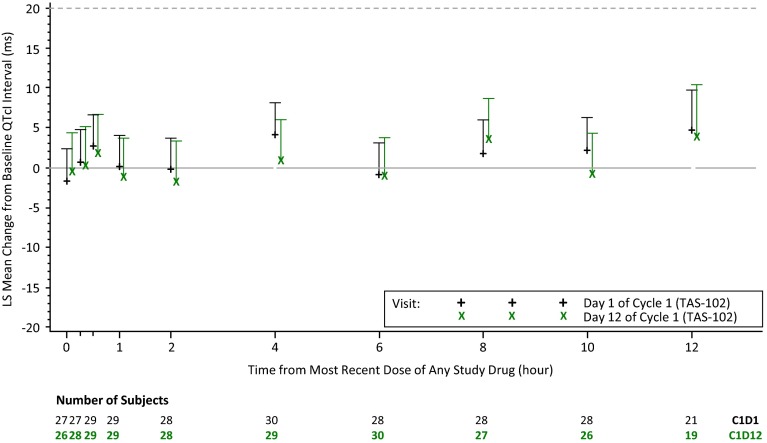
Plot of LS mean and one-sided 95 % CI for placebo-adjusted change from baseline in 12-lead Holter QTcI over time on day 1 and day 12 of cycle 1 (cardiac safety population)

The upper bounds of the one-sided 95 % confidence intervals for the differences in time-matched, baseline-subtracted, 12-lead ECG QTcI intervals between TAS-102 and placebo did not exceed 20 ms at any time point following single-dose (day 1) or multiple-dose (day 12) administration of TAS-102 (Table 2; Fig. 2).

**Table 2:** Comparison of least square mean placebo-adjusted change from baseline in 12-lead Holter QTcI (ms) between TAS-102 and placebo

Day	Postdose hour	TAS-102		Placebo		TAS-102 versus placebo	
		*N*	LS means^a^	*N*	LS means^a^	Difference	90 % CI
1	0	27	−1.9	27	−0.3	−1.6	(−5.6, 2.4)
	0.25	27	−0.9	28	−1.6	0.7	(−3.3, 4.7)
	0.5	29	−1.3	28	−4.0	2.7	(−1.2, 6.6)
	1	29	−2.0	29	−2.2	0.2	(−3.7, 4.1)
	2	28	−0.9	30	−0.6	−0.3	(−4.2, 3.6)
	4	30	2.2	27	−1.9	4.1	(0.2, 8.1)
	6	28	−3.4	29	−2.6	−0.8	(−4.8, 3.1)
	8	28	−1.5	25	−3.3	1.8	(−2.3, 5.9)
	10	28	−1.3	28	−3.5	2.2	(−1.7, 6.2)
	12	21	−0.1	15	−4.9	4.8	(−0.3, 9.8)
12	0	26	−0.9	27	−0.4	−0.5	(−5.6, 4.5)
	0.25	28	−1.3	28	−1.6	0.3	(−4.6, 5.2)
	0.5	29	−1.8	28	−3.6	1.8	(−3.1, 6.7)
	1	29	−3.3	29	−2.1	−1.1	(−6.0, 3.7)
	2	28	−2.1	30	−0.6	−1.5	(−6.3, 4.4)
	4	29	−0.4	27	−1.4	1.0	(−3.9, 5.9)
	6	30	−3.4	29	−2.5	−1.0	(−5.7, 3.8)
	8	27	0.3	25	−3.3	3.6	(−1.5, 8.7)
	10	26	−3.9	28	−3.3	−0.7	(−5.7, 4.3)
	12	19	0.2	15	−3.7	3.9	(−2.5, 10.3)

*LS* least square; *CI* confidence interval

^a^Repeated measures analysis of variance (ANOVA) model: change from baseline in QTcI result = Treatment + Time + Treatment × Time. Compound symmetry covariance was used. Measurements at different time points within each patient’s treatment were treated as repeated measures

Two patients accounted for the changes from baseline in QTcI interval ≥30 ms, one on day −1 and the other at all postdose time points on day 12. One patient had a QTcI interval of 506 ms on a single ECG tracing 4 h after dosing on day 12. The second and third tracings of the protocol-required triplicate tracing were inadequate due to low T-wave amplitude. QTcI intervals on individual tracings at all other study time points were ≤500 ms.

#### QTcF and QTcB

The upper bounds of the one-sided 95 % confidence intervals for the differences in time-matched, baseline-subtracted 12-lead ECG QTcF and QTcB intervals between TAS-102 and placebo also did not exceed 20 ms at any time point on either day 1 or day 12. One patient with a QTcI interval of 506 ms at 4 h postdose on day 12 had a QTcB interval >480 to ≤500 ms at this time point. All other QTcF, QTcB, and uncorrected QT intervals were ≤480 ms for all patients. No patient had a QTcF, QTcB, or uncorrected QT interval >500 ms at any time point.

Four patients had a change from baseline in QTcF interval of ≥30 to <60 ms following multiple-dose administration of TAS-102; five patients had a change from baseline in QTcB interval ≥30 to <60 ms (two following placebo administration and three following multiple-dose administration of TAS-102). Changes from baseline in uncorrected QT intervals of ≥30 to <60 ms were noted for seven patients following placebo administration and six patients each following single-dose and multiple-dose administration of TAS-102. One patient had a change from baseline in uncorrected QT interval ≥60 ms following placebo administration. All other changes from baseline in QTcF, QTcB, and uncorrected QT intervals were <30 ms for all patients.

No significant morphological changes were observed at any time point following placebo administration or single- or multiple-dose administration of TAS-102. Overall, no difference in ECG waveform results was observed with administration of TAS-102 compared with administration of placebo. All T waves and U waves were assessed as normal for all patients.

#### Plasma pharmacokinetics and QTc relation

All 44 patients in the safety population were included in the pharmacokinetic evaluable population for day 1. For day 12, four patients were excluded due to missing assays (2), prohibited concomitant medication (1), or withdrawal of consent (1). Data for the 12-hour time points on days 1 and 12 were excluded for five patients because it appeared that the patients may have received a subsequent dose of TAS-102 before the 12-hour sampling time point.

**Fig. 3:**
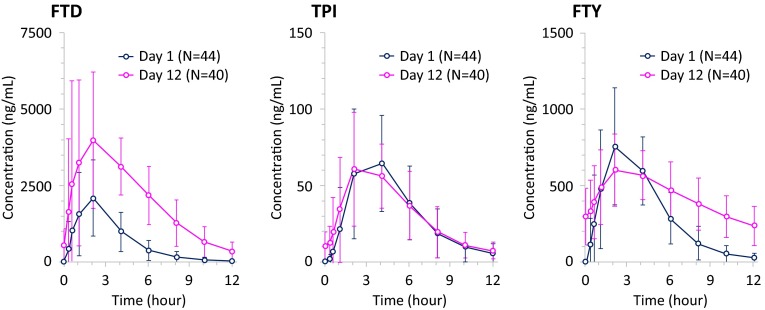
Mean plasma concentration-time profiles after dosing of TAS-102: FTD (PK evaluable population). *N* = 40 for all time points on day 12, except at 12 h postdose (*N* = 39). *FTD* trifluridine, *FTY* trifluoromethyl-2,4(1*H*,3*H*)-pyrimidinedione, *PK* pharmacokinetics, *TPI* tipiracil

Following single-dose administration of TAS-102, the FTD plasma concentration reached a maximum approximately 2 h after dosing and declined with an elimination half-life of approximately 1.5 h (Fig. 3). On day 12, C_max_ of FTD increased approximately twofold and the area under the concentration-time curve from hour 0 to the time of last measurable plasma concentration (AUC_0–last_) increased approximately threefold compared with those on day 1, suggesting an increase in the bioavailability and a decrease in the clearance of FTD after multiple-dose administration of TAS-102. Plasma concentration-time profiles of FTY and TPI were generally similar to those of FTD.

**Fig. 4:**
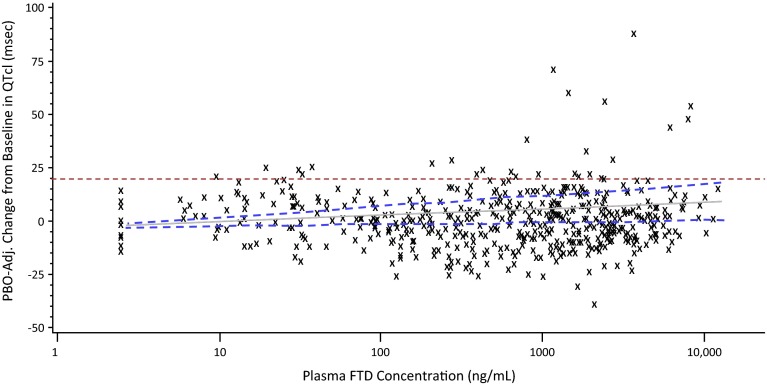
Scatterplot of placebo-adjusted change from baseline in QTcI interval from 12-lead Holter versus observed plasma FTD concentration for all patients (cardiac safety population). *FTD* trifluridine, *PBO* placebo

The slope between the placebo-adjusted change from baseline in QTcI interval and pharmacokinetic concentrations for FTD and TPI was positive as observed from the linear mixed-effect model. However, none of the upper bounds of the one-sided 95 % prediction intervals at mean and observed C_max_ of FTD, TPI, or FTY exceeded the 20 ms non-inferiority margin for QTcI, QTcF, QTcB, and uncorrected QT intervals (Fig. 4). Therefore, the observed pharmacokinetic concentrations of TAS-102 do not suggest a potential for clinically relevant QTc prolongation in this patient population.

However, at most, two placebo-adjusted changes from baseline in QTcI intervals at the time of maximum FTD concentration (1 patient), TPI concentration (2 patients), and FTY (1 patient) were >20 ms. Nothing could be found in the medical history to explain these findings. However, the heart rates of one patient ranged from 63 to 99 bpm during days 1 and 12, and from 69 to 89 bpm for the second patient during day 1 of cycle 1. By comparison, the mean heart rates of the remaining patients ranged from 73.6 to 77.9 bpm during days 1 and 12. No patient experienced ventricular tachycardia or ventricular fibrillation, syncope, or seizure during the study.

## Discussion

Using the same dosing regimen as in the recently completed RECOURSE phase 3 clinical trial [7], TAS-102 caused no clinically relevant prolongation of the QTcI, QTcF, or QTcB intervals. The upper bounds of the one-sided 95 % confidence interval for the largest time-matched mean effect of the drug on the QTc interval excludes 20 ms, which is different from the 10 ms defined by the International Council for Harmonisation, but was felt to be appropriate for this dedicated QTc study in a patient population with advanced solid tumors. Due to the cytotoxic nature of TAS-102, the typical “thorough QT/QTc study” design in healthy volunteers was not applicable. Considering the limitation in the study design versus the potential benefit in a life-threatening indication, the threshold of 20 ms was considered acceptable for this dedicated QTc study in oncology [11].

No cardiac safety concerns had been found in previous clinical trials, although some patients experienced adverse cardiac events, such as atrial flutter or atrial fibrillation [3, 7, 12–14]. One patient in this study experienced a QTcI interval >500 ms on a single tracing following multiple-dose administration, but this could not be confirmed due to low T-wave amplitude on the second and third tracings. There were no morphological changes for T waves or U waves for any patients, and no patient experienced an adverse event of ventricular tachycardia, ventricular fibrillation, or syncope.

Based on the results of the linear mixed-effect model for the relation between plasma FTD, FTY, and TPI concentrations and the placebo-adjusted change from baseline in QTc intervals, no QT-prolonging effect was demonstrated. C_max_ and AUC_0–last_ of FTD increased approximately two- and threefold, respectively, compared with single-dose administration, suggesting an increase in the bioavailability and a decrease in the clearance of FTD with multiple-dose administration of TAS-102. The effect on the QT interval has been shown to be directly related to plasma levels of the drug or its main metabolites [15]. Therefore, in addition to negative results from time-matched analysis of QTcI in TAS-102 compared with placebo, exposure–response analysis, which was based on observed concentration of FTD, FTY, and TPI, also did not show a QT-prolonging effect.

In conclusion, there was no clinically relevant relation between plasma concentrations of TAS-102 and the QTc interval. In addition, TAS-102 had no clinically relevant effects on cardiac repolarization.
